# Specific learning disorders and underlying processes: can WISC-IV subtests predict SLD neurocognitive profiles in Italian children aged 7–14 years?

**DOI:** 10.3389/fpsyg.2026.1934232

**Published:** 2026-09-28

**Authors:** Micaela Capobianco, Miriam Faccio, Carla Giammarco, Dario Camplone, Walter Adriani

**Affiliations:** 1 Department of Economic, Psychological, Communication, Education and Motor Sciences, Telematic University “Niccolo Cusano”, Rome, Italy; 2 "Bimbo Al Centro ETS" Clinical Center, Rome, Italy; 3 Center for Behavioral Sciences and Mental Health, Italian National Institute of Health (ISS), Rome, Italy

**Keywords:** developmental impairments, neurocognitive profiles, school aged children, specific learning disorder, WISC-IV

## Abstract

**Introduction:**

Specific Learning Disorder (SLD) is studied via WISC IV, as well as via neurocognitive impairment profiles arising from clinical tests. However, state-of-the-art appears to be deficitary about the possibility of shared neurocognitive profiles within a given phenotype.

**Objectives:**

We aim to fulfil this gap by investigating, in SLD, formal correlations between WISC IV subtests and nine impairment domains, with their own degrees of severity.

**Participants and methodology:**

160 Italian children, aged 7 to 14 years, have been divided in four groups (Males vs. Females; Primary vs. Lower Secondary School); scores obtained in nine specific domains of abilities have been confronted versus WISC IV scales, using Pearson R correlations, and obtaining 15 × 9 matrices for each group.

**Results:**

The most relevant correlations have been found in the primary-school group only, and in the female group only. The primary-school children presented a strong correlation of WMI with handwriting and reading. On the other hand, in the female group, mental calculation abilities correlated to several subtest indexes, such as: IQ; VCI; WMI. Reading comprehension abilities also correlated to WMI.

**Interpretations:**

A possible explanation to neurocognitive profiles in females is represented by fronto-parietal networks: anomalies in gray-to-white matter ratio, in gray-matter depth, and in white-matter tracts appear to be typical of young females. Results found in the primary-school group underline the developmental role of working memory in learning how to properly read and write. Thus, specific phenotypic areas are implicated in deficitary neurocognitive networks affected in children with SLD.

## 1 Introduction

Specific Learning (SLD) and Attention-Deficit / Hyperactivity (ADHD) disabilities, inasmuch neurodeveloping disorders, have been central over the last decade: a constant process took place of observation and revisitation, for a more complex, dynamic, and applied perspective. Specifically, the necessity of a dimensional approach emerged, aimed to observe their overlapping and comorbidity degree (Astle et al., 2022; Tebartz van Elst et al., 2025).

Within this framework, the contribution of the National Institute of Mental Health (2020), with the beginning of the Research Domain Criteria (RDoC) project in 2008, represented a breakthrough in the conceptualization of neurodevelopmental disorders and mental disorders at developmental age (Insel et al., 2010). RDoC framework, indeed, proposes a dimensional and trans-diagnostic interpretation, grounded in the integration between neurobiological biomarkers and observable behavioral phenotypes (Orsolini et al., 2023a). This perspective is evident in the ways in which SLD has recently been considered by the current ICD-11 (2022) which, compared to the DSM-5 (American Psychiatric Association, 2022), recognizes the need for application, prevention, and intervention purposes. In other words, the need emerged to consider the relationship and interaction between the difficulties encountered by children and their cognitive functions.

Within a complex insight, it is important to observe the relationship between specific learning disabilities and specific cognitive functioning: notably, it is necessary to question whether each learning disorder corresponds to a specific cognitive functioning – which falls into specific underlying processes – or whether, conversely, such disorders can also present independently of a peculiar neuro-psychological profile. From an applied point of view, this issue takes on substantial relevance: in fact, the identification of specific cognitive profiles associated with SLD is of considerable clinical relevance (Capobianco et al., 2025), as it allows for the guidance of neuro-psychological assessment, the early identification of individuals at risk, the personalization of rehabilitation interventions, and the selection of teaching strategies and compensatory tools consistent with the functional profile of the individual child (Gathercole et al., 2008; Behringer et al., 2015). In agreement with multiple cognitive deficits models (Pennington, 2006; van Bergen et al., 2014), a study conducted by Poletti et al. (2018) questioned the categorial approach, highlighting the heterogeneity among learning-impaired children: in this regards, a cluster analysis on a sample of 205 SLD children was carried out, to identify similar cognitive profiles, through the administration of the Wechsler Intelligence Scale for Children – Fourth Edition (WISC-IV) (Wechsler, 2003). Results showed four different cognitive clusters: *Low Verbal*, characterized by weaknesses in verbal subtests (Understanding and Vocabulary) and verbal working memory; *Low Coding*, characterized by difficulties in the Cipher subtest; *Low Executive Functioning*, characterized by deficits in working memory and processing speed; and, finally, *Low Reasoning and Executive Functioning*. The latter represents the profile that underlines the greatest impairment, with critical issues in both fluid reasoning and executive functions. Consequently, within this group great difficulties occur both in mathematics and in reading fluency (Poletti et al., 2018).

Accordingly, a study by Toffalini et al. (2017) supports the presence of specific differences related to cognitive functioning within the diagnostic category of learning disabilities, while recognizing the existence of common profiles that should be considered in the definition. Specifically, the authors argue for the need to introduce a single diagnostic category, thus supporting what indicated by the DSM-5. In their studies, Working Memory Index (WMI) and the Processing Speed Index (PSI), specific to the WISC-IV (Wechsler, 2003), are configured as areas of weakness in all SLD subtypes; consequently, they play a crucial role in the effective acquisition of abilities in different areas of academic learning (Toffalini et al., 2017). Working memory and processing speed relevance in specific learning disabilities has also been confirmed by further studies. The results showed that children diagnosed with SLD scored significantly lower on the Working Memory (WMI) and Processing Speed (PSI) indices than their undiagnosed peers (Malini and Immanuel, 2022; Cornoldi et al., 2014).

Supporting this hypothesis, recent neuroscientific evidence has highlighted how the dorso-lateral prefrontal cortex (dl-PFC) constitutes a key region for cognitive control and executive functioning. In addition to supporting working-memory processes, the dl-PFC contributes to (a) behavior planning, (b) strategy monitoring and updating, (c) goal-oriented action regulation, and (d) complex problem solving (Friedman and Robbins, 2022). Therefore, its impairment can intensify difficulties in maintaining information while reading as well as in problem solving (Flores, 2025). Furthermore, the involvement of posterior parietal regions and temporo-parietal areas contributes to difficulties in visuospatial and linguistic integration. The former plays a central role in spatial information processing, multisensory integration, and visuospatial working memory (Teixeira et al., 2014), while the latter participates in the phonological and semantic processes that support language comprehension and production (Shapleske et al., 1999).

Ultimately, learning disabilities not only depend on the functioning of the cortex, but also involve subcortical regions; in this regard, some researchers have found that, in participants with dyscalculia and dyslexia, pathways followed by white matter are compromised (Ashkenazi et al., 2013). Specifically, these are alterations of the white matter pathways, particularly of the arcuate fasciculus, the superior longitudinal fasciculus, and the inferior longitudinal fasciculus, which connect the temporo-parietal and occipito-temporal regions (involved in phonological, visual, and semantic processing). Impairment of such connections can hinder the synchronization of cognitive processes necessary for reading and school learning (Kucian et al., 2014; Skeide, 2018).

Concurrently, more recent evidence calls into question the diagnostic and predictive capacity of the cognitive profile assessed through the WISC-IV (Wechsler, 2003). In particular, a study conducted on children diagnosed with developmental dyscalculia has proven that children with severe mathematics difficulties exhibit lower global cognitive functioning than children with learning disabilities without impairment of the logical-mathematical area, but cannot be identified based on the multivariate WISC-IV index model.

### 1.1 State of the art

Specific Learning Disabilities (SLD) present variable incidence rates according to age and geographical area. Globally, according to international diagnostic manuals, such as the DSM-5 and ICD-11, the prevalence of SLD in school-aged children fluctuates between 5 and 15% of the population; males to females ratio varies from 1.5:1 to 3:1. In Italy, according to the Statistics Office of the Ministry of Education and Merit (2024), the percentage of students with SLD was 6% in the 2022/2023 school year (Ministry of Education and Merit, 2024). Over the last 13 years, the percentage of students has increased from 0.9 to 6.0% (Ministry of Education and Merit, 2024). To understand the phenomenology of the disorder, it is essential to analyze gender-based cognitive functioning. However, a review of the scientific literature in this field presents important methodological limitations. Most research, in fact, has compared males and females based exclusively on performance at the observable level (that is, on manifest variables, such as summary scores on the Wechsler scales), rather than adopting a latent-variable model. This overt indicator approach amplifies risks of producing a bias, as global scores result from a weighted combination of subtests in which one gender can excel over the other, masking the true structure of cognitive ability (Hunt, 2010). Although the official WISC-IV manual (Wechsler, 2003) reports several confirmatory factor analyses to support the latent structure of the instrument, it does not provide any information on any gender differences.

A research conducted by Pezzuti and Orsini (2016) on an Italian normative sample of 2,200 typically developing participants revealed specific divergences through the examination of latent means: males obtained higher scores on the Verbal Comprehension Index; on the other hand, females had a better performance on the Processing Speed Index (Pezzuti and Orsini, 2016). A recent meta-analysis conducted by Esposito and Giofrè (2025) examined gender differences in cognitive profiles assessed by the WISC-IV. The results showed no significant differences in either Total Intellectual Quotient (IQ) or fluid reasoning skills. However, males showed slightly higher scores in verbal comprehension skills, particularly in the Information, Vocabulary, and Comprehension subtests, as well as in visuospatial and visuo-perceptual skills, assessed through subtests such as Block Design and Visual Puzzles. In contrast, females performed better in processing speed, with a particularly evident advantage in the Coding subtest. Regarding working memory, less unequivocal results emerged: males reported slightly higher scores in the Arithmetic subtest, while females showed a slight advantage in the Digit Span subtest, although this difference did not reach statistical significance.

Despite this evidence, providing a clear picture for the typically developing population, the scientific literature appears insufficient as far as children with SLD are concerned. To fill this gap, Giofrè et al. (2022) examined WISC-IV cognitive profiles in 537 females and 699 males, aged between 7.1 and 16.9 years, diagnosed with SLD. Results showed female superiority regarding Working Memory Index and Elaboration Speed Index. Moreover, confronting profiles of children with dyslexia, dyscalculia, and mixed SLD, results corroborated how children with different SLD diagnoses were prone to different profiles, both within and across categories, and that sex differences between subtests and indexes appeared in only some, but not all tests (Giofrè et al., 2022).

As required by the previously cited ICD-11 and DSM-V (American Psychiatric Association, 2022), it is not possible to diagnose SLD in the presence of an intellectual development disorder. For this reason, the cognitive assessment carried out through the WISC-IV (Wechsler, 2003) plays a major role, as learning difficulties manifest specifically only in the presence of typical levels of intellectual functioning, that is, an IQ score equal to at least 70 (American Psychiatric Association, 2022). Additionally, as highlighted by the Consensus Conference promoted by the *Istituto Superiore di Sanità* (ISS, Lorusso et al., 2014), the IQ criterion should not be interpreted in a rigidly exclusionary way for the diagnosis of SLD. In particular, an IQ score below 85 does not automatically exclude the presence of a specific learning disability, since academic difficulties and neuro-psychological profiles may be overlapping on those observed in participants with normal IQ, making a clinical assessment (of the overall functioning profile) more appropriate (Lorusso et al., 2014). Cornoldi et al. (2014) confronted cognitive profiles in children with SLD and with Intellectual Disability, paying close attention to SLD cases associated with borderline cognitive functioning. The results showed that children with borderline SLD and IQ had a relatively higher General Ability Index (GAI) than the Cognitive Competence Index (ICC), suggesting that their Total IQ may have been underestimated by difficulties in working memory and processing speed. The authors hence highlighted how, in these cases, GAI may represent a more accurate measure of general cognitive abilities than Total IQ (Cornoldi et al., 2014).

Adaptive functioning and intellectual abilities may have broader implications on adaptive and behavioral outcomes throughout development. Research on intellectual disability highlighted the relevance of intellectual and psychosocial disadvantages in relation to involvement in the criminal justice system and legal decision-making processes (Giannouli, 2020). Adolescents with SLD are especially more likely to engage in delinquent behavior and juvenile offenses than adolescents without disabilities (Shandra and Hogan, 2012). However, the nature and direction of the relationship between intellectual disability and delinquent behavior remains a matter of debate today. In summary, although SLD and intellectual disability represent distinct diagnostic conditions, the current evidence highlights the importance of examining cognitive profiles in SLD beyond assessing academic performance alone.

### 1.2 Objectives and endpoints

Our research fits within the framework of the above-illustrated studies, as it aims to understand whether and how the various indices and subtests that compose the WISC-IV (Wechsler, 2003) can roughly highlight a correlation with the various facets of a specific learning disability: this kind of data issued by our own observations is of considerable clinical relevance, as it allows for the design and planning of more targeted and effective interventions. Specifically, the present study examines *nine* learning areas: reading for both correctness and speed, reading comprehension, handwriting, dysorthography, written text production, numeracy, numerical skills, and problem solving. Based on the reference literature, we hypothesize the existence of significant associations between WISC-IV cognitive indices (Wechsler, 2003) and performance in the nine school-relevant learnings. The data analysis aims to understand which cognitive dimensions may be most involved in different school skills. These profiles were considered according to developmental age, distinguishing two different stages: two sample groups were selected based on the class attended by the children at the time of diagnosis, i.e., primary school *versus* lower secondary school. Furthermore, the gender factor was examined, with the hypothesis of identifying possible significant differences between males *versus* females in cognitive profiles and academic performance.

Especially, the Processing Speed Index (PSI) and the Working Memory Index (WMI) are expected to directly influence general learning skills, plus specifically those related to logical-mathematical skills. As regards the Verbal Comprehension Index (VCI), it is assumed that this has a predominant role in the comprehension of written text and in the written production of the text. A further hypothesis concerns the role of the Total Intellectual Quotient (Total IQ), which is expected to be more closely linked to verbal and comprehension skills. Finally, the study explores the possible presence of gender differences in patterns of association between cognitive profile and learning, with the aim of contributing to a more nuanced and clinically relevant understanding.

This study aims to offer an innovative approach and relevant contribution in several respects. First, it is based on empirical data from a real clinical archive, consisting of evaluations carried out in ecological diagnostic contexts, and not on pre-selected samples. This approach allows to start from direct observation of data, and inductively trace the relationships between cognitive profiles and learning difficulties, from a perspective that proceeds from empirical data to theoretical constructs. Second, the study systematically analyzes the correlations between specific WISC-IV indices and subtests *versus* a broad spectrum of academic skills, allowing us to highlight differential patterns that are unlikely to emerge through less comprehensive approaches. Finally, the integration of variables related to schooling and gender enables us to explore the possible existence of distinct cognitive profiles within the clinical population, contributing to a more structured and dimensional view of the disorder. In summary, this work is configured not only as a correlation study, but as an attempt to reconstruct the cognitive processes underlying SLD, starting from real clinical data, and with potential new theoretical and applied implications.

## 2 Materials and methods

### 2.1 Participants

The clinical sample was selected from a database archive, based on empirical data collected at the Health Association “Bimbo al Centro ETS” in Rome, which offers a clinical assessment and developmental intervention services.

Specifically, the archive of children diagnosed with SLD was retrospectively analyzed.

Based on archived medical records, relating to assessments conducted between 2019 and 2025, a total of 160 subjects have been included in the study. All participants had been diagnosed with SLD according to the ICD-10 classification system, including all of the following diagnostic categories:

- Specific Reading Disorder (F81.0)
- Specific Spelling Disorder (F81.1)
- Specific Arithmetic Skills Disorder (F81.2)
- Mixed Disorder of Scholastic Skills (F81.3)

Subsequently, the sample was divided into two groups based on educational level at the time of diagnosis: 80 participants diagnosed during the primary school (mean age = 8.6 years) *versus* 80 during the lower secondary school (mean age = 12.3 years).

Furthermore, in order to examine any gender differences, separate analyses were conducted for males (*n* = 95) and females (*n* = 65), regardless of school educational level.

It was not possible to control for the socio-cultural and socio-economic level of the families; therefore, the sample showed some heterogeneity with respect to these variables, which may have influenced, at least in part, cognitive functioning and performance on learning tests. To be included in the study, participants had to meet the following criteria: an Intellectual Quotient (IQ) equal to or greater than 80, assessed by the WISC-IV (Wechsler, 2003; Italian adaptation by Orsini et al., 2012); birth between the 35th and 41st week of gestation, including any cases of mild prematurity not associated with clinically ascertainable neurological damage; absence of major psychiatric or neurological disorders; absence of diagnosed autoimmune diseases; absence of febrile or infectious states occurring at evaluation or within the 2 weeks preceeding the evaluation. All diagnoses were made according to ICD-10 criteria and in line with the recommendations of the Consensus Conference published later, as guidelines, by the Istituto Superiore della Sanità (2022).

### 2.2 Evaluation of learning domains

In order to get a homogeneous sample, reports presenting the same test batteries capable of evaluating learning domains were selected. Regarding reading skills, both in terms of accuracy and fluency, as well as reading comprehension, assessments were based on the MT-3 Clinical battery – *The assessment of reading and comprehension skills for primary and lower secondary school* (Cornoldi and Carretti, 2016). For the assessment of spelling, graphic skills and written production, reference was made to the performance obtained by administering the BVSCO-3 – *Battery for the clinical assessment of writing and spelling skills* (Cornoldi et al., 2022); limited to primary school, we used also the BHK – *synthetic scale for the assessment of writing at developmental age* (Di Brina and Rossini, 2021). Lastly, two different batteries were used to evaluate logical-mathematical skills: the BDE-2 – *Battery for developmental dyscalculia* (Biancardi et al., 2016), as well as the AC-MT-3 for 6–14 years – *Clinical tests: tests for evaluating calculation skills and mathematical reasoning* (Cornoldi et al., 2020).

Regardless of the different features of the two batteries, communal tests have been selected. Namely, to evaluate the *numerical area*, i.e., the ability to comprehend the numerical code, and the syntactic and semantic number structures, we included only individuals to whom *Number Writing* test from BDE-2 battery, and *Dictation of Numbers* from AC-MT 3 battery, had been administered. To evaluate the ability to *mentally manipulate quantities*, *Mental Calculation* has been considered, as it appears in both tests. Lastly, regarding *problem solving* abilities, *Approximative Calculation* test has been examined, also shared between the two batteries, as this competence is based on the correct functioning of the so-called “approximate number system”.

Of note, the latter states that arithmetic estimation processes do not arise from random intuitive responses, but rather from the active application of sophisticated heuristic and rounding strategies (Ganor-Stern, 2016). The author’s research has, in fact, demonstrated how the choice to approximate a complex calculation requires a cognitive flexibility which is similar to problem solving: in brief, the participant must analyze the numerical structure of the stimulus, to select the most efficient estimation strategy and exclude all unlikely results.

Statistical analyses have been conducted considering the following *nine* parameters: reading accuracy; reading fluency; reading comprehension; handwriting; spelling (taking into account the total errors); written production (assessed by errors percentage); three mathematical skills such as number area, mental calculation, and problem solving, assessed by the correctness parameter.

### 2.3 Quantitative analysis of learning deficits

To allow for quantitative analysis of the results, performances detected through instruments were converted into ordinal scores. For the MT-3 and BVSCO-3 tests, a four-level scale was adopted: 3 = criterion fully achieved; 2 = sufficient performance; 1 = request for attention; 0 = request for immediate intervention. For the BDE-2 and AC-MT-3 scales, scores were assigned on the basis of the percentile bands predicted by the standardized tests: 0 = clinical band (≤5° percentile); 1 = below standard (6°-24° percentile); 2 = within the standard (25°-74° percentile); 3 = above standard (≥75° percentile). Finally, handwriting was assessed through a qualitative analysis based on the impairment of the graphic line, posture assumed by the children, and sheet space organization; the following scores were then assigned: 3 = sufficient; 2 = mild; 1 = moderate; 0 = severe dysgraphia.

### 2.4 Statistical analysis

Analyses’ final product is represented throughout four correlation matrices, one for each clinical group, structured as a double-entry table, in which rows correspond to the WISC-IV scales and columns to the learning domains (the nine parameters obtained from medical records).

Each cell of the matrix contains the Pearson linear correlation coefficient (R), calculated between the two scores, obtained by the same individual in the specific WISC scale (row) *versus* the corresponding learning parameter \ domain index (column). Pearson’s R coefficient values range between +1 and −1: values close to +1 indicate a high positive correlation (as one variable increases, the other also increases), while values close to −1 indicate a high negative correlation (as one variable increases, the other decreases). A value of zero indicates no linear relationship between the variables. From a graphical point of view, a correlation equal to +1 or −1 implies that the data are arranged exactly along a line (respectively, increasing or decreasing), while intermediate values describe a scatter cloud, approximately elliptical, oriented depending on the direction of the regression line, which describes the relationship.

Because of the large number of comparisons made (given by the product between the number of WISC scales and the number of learning parameters \ domains indices: 15 × 9 = 135 R-values), it has appeared necessary to adopt a more restrictive criterion for statistical significance, inspired by the well-known Bonferroni correction, in order to reduce the risk of false positives. Hence, significance level has been set at *p* < 0.001, corresponding to a 0.1% probability of error. On a total number of comparisons greater than one hundred, this threshold allows the global risk of false positives to be kept within acceptable limits.

The analyzed sample consisted of *N* = 160 participants (*n* = 80, for each age group), corresponding to 79 degrees of freedom. Based on these parameters, the cutoff value of the correlation coefficient for statistical significance (*p* < 0.001) has been found to be R ≥ 0.359. In the matrices of the two age groups (i.e., school classes), all cells containing R values greater than +0.359 or less than −0.359 were considered statistically significant and appropriately highlighted, while the remaining non-significant cells were not emphasized and ignored.

Furthermore, to optimize the analysis of gender differences, the whole sample was divided into males and females, regardless of age. The male sample equals *n* = 95, while the female sample is composed of *n* = 65 individuals. For this reason, the degree of freedom in males is equivalent to 94, and the threshold value is R ≥ 0.3307; Females instead have 64 degrees of freedom, therefore the cutoff value is R ≥ 0.3959; in agreement with Cohen “d,” detected effects in our approach are “moderately strong” in size.

Following Pearson correlation test, a Spearman correlation test has been also performed. This choice is due to the fact that, for the nine SLD learning domains, characters’ modalities are of the ordinal \ discrete numerical type. Furthermore, this double approach allows us to validate or confute what found to be significant through the calculation of Pearson’s R-value.

To calculate the threshold value of Spearman’s ρ (rho), a *p* value <0.001 was set, which resulted in a minimum threshold value of 0.364; this means that Spearman’s ρ yields significance for values greater than the established Pearson’s R-value. For the two gender-based groups, a Spearman’s ρ threshold of 0.3348 was calculated for the group of male students, and a threshold of 0.4009 for the group of female students. Subsequently, the significancy emerging with Pearson R-values was compared with the significancy obtained through Spearman’s ρ values; establishing that, whereas correlations obtained with the Pearson test are not also confirmed fully with the Spearman test, the Pearson correlations are not to be considered reliable.

## 3 Results

### 3.1 Common correlations to all four matrices

The ability READING COMPREHENSION showed the most abundant quantity of correlation with many WISC-IV subscales. Of course, it showed a significant correlation with the WISC-IV subscale named TOTAL IQ for the group primary school (R = 0.5541, *p* < 0.05), the group lower secondary school (R = 0.5153, *p* < 0.05), the group female children (R = 0.6511, *p* < 0.05) and the group male children (R = 0.4454, *p* < 0.05) (Table 1).

**Table 1:** Reading comprehension: its correlations in the four groups. WISC items reported in the columns; R-values in the cells.

Group displaying significant correlations	Total IQ	VCI	Vocabulary	Verbal comprehension	Letter number sequencing
Primary matrix	0.5541	0.5582	0.5654	0.4840	0.4362
Lower secondary matrix	0.5153	0.5345	0.4707	0.4996	0.3950
Females matrix	0.6511	0.5923	0.5509	0.4768	0.4939
Males matrix	0.4454	0.5130	0.4864	0.5017	0.3595

The same ability READING COMPREHENSION showed also a very expected significant correlation with the WISC-IV subscale named VERBAL COMPREHENSION INDEX (VCI) for the group of primary school (R = 0.5582, *p* < 0.05), the group lower secondary school (R = 0.5345, *p* < 0.05), the group female children (R = 0.5923, *p* < 0.05) and the group male children (R = 0.5130, *p* < 0.05). Another significant correlation of READING COMPREHENSION was with the WISC-IV subscale named VOCABULARY for the group primary school (R = 0.5654, *p* < 0.05), the group lower secondary school (R = 0.4707, *p* < 0.05), the group female children (R = 0.5509, *p* < 0.05) and the group male children (R = 0.4864, *p* < 0.05).

The final significant correlation of READING COMPREHENSION was with the WISC-IV subscale named LETTER AND NUMBER SEQUENCING for the group primary school (R = 0.4362, *p* < 0.05), the group lower secondary school (R = 0.3950, *p* < 0.05), the group female children (R = 0.4939, *p* < 0.05), and the group male children (R = 0.3595, *p* < 0.05).

The first three WISC-IV subscales listed above showed the strongest Pearson R value (all > 0.48). The last two WISC-IV subscales showed the less strong R values (all < 0.50 but one).

### 3.2 Common correlations in at least three matrices

The ability READING COMPREHENSION showed a significant correlation with the WISC-IV subscale named WORKING MEMORY INDEX (WMI) for the group primary school (R = 0.4774, *p* < 0.05), the group female children (R = 0.5073, *p* < 0.05) and the group male children (R = 0.3468, *p* < 0.05), but not for lower secondary school (not far from significance, R = 0.3534) (Table 2).

**Table 2:** Reading comprehension: its correlation in three out of four groups. WISC items reported in the columns; *R*-values in the cells.

Group displaying significant correlations	WMI	Similarities
Primary matrix	0.4774	
Lower secondary matrix		0.3989
Females matrix	0.5073	0.4206
Males matrix	0.3468	0.3425

The same ability showed also a significant correlation with the WISC-IV subscale named SIMILARITIES for the group lower secondary school (R = 0.3989, *p* < 0.05), the group female children (R = 0.4206, *p* < 0.05) and the group male children (R = 0.3425, *p* < 0.05), but not primary school (not far from significance, R = 0.3506). The fact that a fourth group was however very close to the threshold suggests that with a greater number of patients also the fourth group would yield a significance.

### 3.3 Common correlations to at least two matrices

The ability ACCURACY showed a significant correlation with the WISC-IV subscale named WORKING MEMORY INDEX (WMI) for the group of primary school (R = 0.4474, *p* < 0.05) and the group male children (R = 0.3639, *p* < 0.05), but not for lower secondary school and female children (Tables 3–5).

**Table 3:** Accuracy: correlations. WISC items reported in the columns; *R*-values in the cells.

Group displaying significant correlations	WMI
Primary matrix	0.4474
Males matrix	0.3639

**Table 4:** Fluency: correlations. WISC items reported in the columns; R-values in the cells.

Group displaying significant correlations	Digit span	WMI
Primary matrix	0.4079	
Lower secondary matrix		0.3814
Males matrix	0.3982	0.3788

**Table 5:** Reading comprehension: correlations. WISC items reported in the columns; *R*-values in the cells.

Group displaying significant correlations	Digit span
Primary matrix	0.4026
Females matrix	0.4326

The ability FLUENCY showed a significant correlation with the WISC-IV subscale named DIGIT SPAN for the group primary school (R = 0.4079, *p* < 0.05) and the group male children (R = 0.3982, *p* < 0.05), but not for lower secondary school and female children. It also showed a significant correlation with the WISC-IV subscale named WORKING MEMORY INDEX (WMI) for the group secondary lower school (R = 0.3814, *p* < 0.05) and the group male children (R = 0.3788, *p* < 0.05), but not for primary school and female children.

The ability READING COMPREHENSION showed a significant correlation with the WISC-IV subscale named DIGIT SPAN for the group primary school (R = 0.4026, *p* < 0.05) and the group female children (R = 0.4326, *p* < 0.05), but not for lower secondary school and male children. Here, we see for the first time a differential effect, which will then explode when single-group correlations are concerned. Normally, when a mathematical skill is considered, its correlation concerns only the primary school students and females.

### 3.4 Significant non-common correlations

The ability ACCURACY shows a significant correlation with the WISC-IV subscale named DIGIT SPAN (R = 0.4257, *p* < 0.05) only for the primary school group (Table 6).

**Table 6:** Non-common correlations. Group showing correlation reported in the columns; *R*-values in the cells. Empty cells = NS.

Domain indexes (left) and (right) WISC items showing significant correlations	Primary matrix	Lower secondary matrix	Females matrix
Accuracy and digit span	0.4257		
Handwriting and WMI	0.4303		
Handwriting and digit span	0.3836		
Handwriting and letter number sequencing	0.3726		
Spelling and WMI	0.5055		
Spelling and digit span	0.4872		
Spelling and letter number sequencing	0.4061		
Math calculation and total IQ			0.5292
Math calculation and VCI			0.4221
Math calculation and WMI			0.3999
Math calculation and letter number sequencing			0.4289
Math calculation and similarities			0.4533
Problem solving and WMI			0.4184
Problem solving and digit span			0.4251
Comprehension and perceptual reasoning			0.4112
Problem solving and coding		0.3167	
Comprehension and matrix reasoning		0.3776	

The ability HANDWRITING shows a significant correlation with three WISC-IV subscales: WORKING MEMORY INDEX (WMI) (R = 0.4303, *p* < 0.05), DIGIT SPAN (R = 0.3836, *p* < 0.05), and LETTER-NUMBER SEQUENCING (R = 0.3726, *p* < 0.05), only for the primary school group.

The ability SPELLING shows a significant correlation with the WISC-IV subscales named WORKING MEMORY INDEX (WMI) (R = 0.5055, *p* < 0.05), DIGIT SPAN (R = 0.4872, *p* < 0.05), and LETTER-NUMBER SEQUENCING (R = 0.4061, *p* < 0.05) only for the primary school group. It is quite evident that correct handwriting and correct spelling when reading are related to working memory, but also to two mathematical / logic WISC-IV scales, and this only in younger students aged less than 10 years.

The ability MATH CALCULATION shows relevant correlation with the WISC-IV subscales named TOTAL IQ (R = 0.5292, *p* < 0.05), VCI (R = 0.4221, *p* < 0.05), WORKING MEMORY INDEX (WMI) (R = 0.3999, *p* < 0.05), LETTER-NUMBER SEQUENCING (R = 0.4289, *p* < 0.05), and SIMILARITIES (R = 0.4533, *p* < 0.05) only for the female children group.

The ability PROBLEM SOLVING shows relevant correlation with the WISC-IV subscales named WMI (R = 0.4184, *p* < 0.05) and DIGIT SPAN (R = 0.4251, *p* < 0.05) only for the female children group. It is indeed a striking finding that the same correlation was completely absent in the male gender.

Another significant correlation is between the ability PROBLEM SOLVING and the WISC-IV subscale named CODING (R = 0.3617, *p* < 0.05), only for the lower secondary school group. The last significant correlation is between the ability COMPREHENSION and the WISC-IV subscale PERCEPTUAL REASONING INDEX (PRI) (R = 0.4112, *p* < 0.05) only for the female children group, and the WISC-IV subscale MATRIX REASONING (R = 0.3776, *p* < 0.05) for lower secondary school group.

### 3.5 Additional Spearman\Pearson considerations

This analysis revealed how some of the emerging correlations from the Pearson test were not confirmed as significant in the Spearman test. Particularly, correlations whose Pearson R value was significantly higher than that of Spearman ρ were therefore discarded (Table 7).

**Table 7:** Comparison between correlations emerged from Pearson and Spearman tests.

Correlation	Group	R-Pearson	Rho-Spearman	Difference
Hand writing vs. Letter number sequencing	Primary school	0.3726	0.3476	- 0.0251
Comprehension vs. Matrix reasoning	Lower secondary school	0.3776	0.2968	- 0.0808
Comprehension vs. Perceptual reasoning	Female children	0.4112	0.3366	- 0.0746
Comprehension vs. Digit span	Female children	0.4326	0.3739	- 0.0587
Math calculation vs. VCI	Female children	0.4221	0.3748	- 0.0473
Math calculation vs. WMI	Female children	0.3999	0.3785	- 0.0213

## 4 Discussion

The present research aimed to investigate the relationships between WISC-IV indices and different school skills, impaired in Specific Learning Disabilities (SLD), taking into account gender and schooling variables. Based on the scientific literature, several hypotheses were formulated: the existence of significant associations between WISC-IV indices and learning domains; a correlation between WMI as well as PSI and logical-mathematical skills; a predominant involvement of VCI in reading comprehension and production processes; the existence of a relationship between total IQ and verbal skills; and lastly, the presence of gender differences in correlation patterns.

Obtained results only partially confirm these hypotheses. In line with the first hypothesis, several significant correlations emerged between WISC-IV indices and academic skills, supporting a multidimensional model of SLD. However, these associations are not uniform across groups, suggesting that cognitive functioning in SLD cannot be interpreted in a unified way, but must be considered in relation to specific evolutionary trajectories and individual differences.

### 4.1 Phenotype-psychometrical considerations

Contrary to expectations, no significant correlations emerged between Processing Speed Index (PSI) and learning abilities. However, a positive correlation was observed between problem solving ability and the Coding subtest in the lower secondary school group. Similar results were highlighted by the study by Kubas et al. (2014), who underlined a predictive relationship between the Coding test and logical-mathematical skills.

On the other hand, as expected, WMI was found to be related to numerous learning skills, being involved in reading, writing and logical-mathematical skills. In reading, it is implicated in both accuracy and fluency parameters, in the primary-school and male groups. However, it continues to affect fluency in the middle school group as well. These data depict a greater involvement of working memory in primary school, highlighting the need to interpret this relationship from a developmental perspective. Indeed, as per the state-of-the-art (Peng et al., 2018; Vasileiou, 2026), younger children have not yet automated reading and writing processes and must, therefore, allocate greater working memory resources to simultaneously manage coding, procedure acquisition, and verbal information processing. Recently, it has been shown that working-memory deficit in children with reading impairment is associated with alterations in resting-state functional connectivity of several brain networks: the Default Mode Network (DMN), consisting of the posterior cingulate cortex, prefrontal cortex, angular gyrus, and middle temporal cortex; the Salience Network (SN), comprising the anterior cingulate cortex, anterior insula, the supramarginal gyrus and rostral prefrontal cortex; and the Fronto-Parietal Network (FPN), which includes the dorso-lateral prefrontal cortex and the posterior parietal cortex (Fang et al., 2016; Flores-Gallegos et al., 2024). In light of this evidence, it is possible to hypothesize that reduced performance on WISC-IV Letter-Number Sequencing subtest may reflect an alteration in the functioning of the Default Mode Network, the Salience Network, and\or the Fronto-Parietal Network.

Additionally, those subtests composing WMI, namely Digit Memory and Letter-Number Sequencing, are involved in various skills. For example, the Letter-Number Sequencing correlates with reading comprehension in all groups; in contrast, Digit Span and reading comprehension emerge only in primary-school and females. This finding is confirmed by a study conducted on primary school children diagnosed with SLD, which demonstrated that enhancing working memory leads to an improvement in reading-comprehension skills (Okur and Aksoy, 2025). Furthermore, it has been shown that working memory does not appear to directly improve reading comprehension; instead, it acts indirectly. In particular, working memory affects decoding accuracy, which for its part affects text comprehension. Thus, working memory mainly influences the initial phase of reading and indirectly the understanding of meaning (Orsolini et al., 2023b).

As regards the writing skills, WMI plus (specifically) the Digit Span subtest showed a correlation with handwriting in the primary-school group, suggesting a greater involvement of working memory at this stage of development. Similarly, spelling also shows correlations with WMI and with Digit Span as well as Letter and Number Sequencing subtests, corroborating the central role of working memory in writing processes. A study conducted by De Vita et al. (2021) highlighted that, in writing fluency tests and in more complex tasks that require not only good phoneme-grapheme conversion skills, but also good access to orthographic knowledge, active components of working memory are crucial.

A noteworthy result concerns gender differences. Several correlations emerged exclusively in the female group, while they were not observed in males. Notably, working memory appears to take on a more relevant role in logical-mathematical skills. In problem solving skills, a correlation emerges especially with Digit Span subtest, suggesting that, in the examined sample, females rely more on working memory resources than males. Mental calculation is also associated with several cognitive indices: total IQ, Similarities subtest, as well as Letter and Number Sequencing subtest. These results suggest that total IQ, VCI, and WMI may represent the main predictors of numeracy skills in the female group. Some studies on parietal lobe morphology have shown that women and girls possess a higher gray-to-white matter ratio than males (Koscik et al., 2009; Salinas et al., 2012). Furthermore, Sowell et al. (2007) observed a greater proportional depth of gray matter in girls, particularly in the right inferior parietal lobe and posterior temporal regions. This evidence could help explain the results that emerged in the present study, in which numeracy skills show strong linkage with total IQ (Capobianco and Costa, 2024), verbal understanding (VCI), Working Memory Index (WMI) and related WISC-IV subtests in the female group.

Ultimately, reading comprehension represents the most associated skill with cognitive indices and related subtests, showing correlations in all analyzed groups. Specifically, it correlates with total IQ and Verbal Comprehension Index (VCI), especially through the Vocabulary subtest, which assesses lexical richness, knowledge of word meaning, and verbal expression ability. The Similarities subtest, also belonging to VCI, is instead related to reading comprehension exclusively in the lower secondary (i.e., middle school) group, both in males and females. The correlation between the Vocabulary subtest and treading comprehension was also found in a study conducted by Guimarães and Mousinho (2019), who state that reading comprehension requires a vocabulary demonstrating a deeper level of semantic knowledge, beyond simply reading words. Furthermore, many individual differences in comprehension skills can be explained by vocabulary breadth, vocabulary depth, and strength between lexical representations. For this reason, to improve understanding it is important to work on a strengthening of the vocabulary, that is, the lexicon: i.e., by increasing the number of representations it contains; and by improving the quality and strength of connection of these representations (Swart et al., 2017). Overall, reading comprehension is the skill with the greatest number of associations with WISC-IV cognitive indices, confirming its position as the skill most influenced by general cognitive functioning.

### 4.2 Neurobiological considerations

From a neuroscientific perspective, alterations in the gray-to-white matter ratio (GWR), paired with abnormalities of the white matter tracts, appear to represent common characteristics to the different Specific Learning Disabilities, as do alterations in functional connectivity, both in terms of hyperconnectivity and hypoconnectivity, between different brain regions (Jolles et al 2016; Flores-Gallegos et al., 2024). Particularly, a neural network comprising the fronto-parietal regions, including the intra-parietal sulcus and the dorso-lateral prefrontal cortex, is involved in numerous cognitive difficulties observed in children and adolescents with SLD.

A study conducted by Dehaene et al. (2005) highlighted three parietal circuits involved in number processing: the horizontal segment of the intraparietal sulcus (HIPS), the left angular gyrus, and the bilateral posterior superior parietal system. Specifically, the former is systematically activated whenever numbers are manipulated, regardless of their notation, with an increasing in activation the more the task focuses on processing quantities; the second one supports the manipulation of numbers in verbal form; the latter is involved in orienting attention along the mental number line (Dehaene et al., 2005). It is also important to emphasize how the left angular gyrus has been associated not only with verbal numerical processing processes, but also with broader skills related to number management in everyday life, such as financial capacity throughout one’s life span. In particular, evidence from studies on individuals with mild amnestic cognitive impairment (aMCI) shows that financial capacity is significantly related to left angular gyrus volumes, as well as limbic structures such as the right amygdala, suggesting an integration between arithmetic and emotional-decisional components in economic processes (Giannouli and Tsolaki, 2019). In a longitudinal study, this relationship is maintained over time, indicating that structural changes in these brain areas can directly impact financial literacy.

Further data from structural neuroimaging studies highlight how, in individuals with anamnestic cognitive impairment (CMI), performance in financial skills is moderately correlated with the volume of the angular gyrus and precuneus, and how the angular gyrus represents a significant predictor of financial capacity measured by standardized instruments (Griffith et al., 2010). Mediation analyses further suggest that this relationship is partially explained by arithmetic skills and attentional processes, indicating how the contribution of the angular gyrus to money management runs through basic cognitive functions but also through more complex mechanisms of cognitive integration.

Overall, this evidence suggests that the left angular gyrus plays a crucial role in applied and functional numerical cognition, extending its contribution beyond symbolic number processing to the skills needed to tackle ecologically-sound tasks, such as economic resource management. This set of data reinforces the hypothesis of marked heterogeneity in Specific Learning Disabilities and supports dimensional interpretative models (Lunardon et al., 2023).

### 4.3 Research limitations and future perspectives

The clinical sample was obtained from empirical data collected within the clinical activity of an association specialized in the evaluation and treatment of neurodevelopmental disorders. The clinical and retrospective nature of data collection necessarily constrained the available variables to those that had been systematically detected in clinical practice. For this reason, this data collection method may constitute a first limitation of the research, as it was not possible to integrate further information or retrospectively introduce new variables not collected at the time of the evaluation. All available data relevant to the study objectives were therefore included in the analyses. This characteristic must be considered when interpreting the results; more parameters need to be explored further in future studies, through prospective and more structured protocols, which allows for the systematic collection of additional cognitive, clinical, anamnestic, and socio-cultural information relating to the rehabilitation interventions received.

Although the sample is relatively large and allows for a distinction between individuals attending primary and lower secondary school, further studies should investigate the associations between underlying cognitive processes and different types of SLD. In particular, it may be interesting to distinguish between the cognitive profiles associated with reading, writing, and numeracy difficulties, in order to verify the possible presence of specific functional patterns and to clarify whether different cognitive mechanisms characterize different SLD profiles. To achieve this, future studies could use larger samples and more advanced analytical approaches, such as cluster analysis, to identify any subgroups characterized by specific associations between cognitive processes and learning difficulties.

A further limitation concerns the heterogeneity of socio-cultural and clinical conditions of the individuals. Indeed, the presence of previous clinical diagnoses, such as Language Development Disorder or Motor Coordination Disorder, may have contributed to the observed variability in cognitive outcomes and learning. For this reason, subsequent research should consider these variables and investigate deeper into the impact of specific anamnestic factors on the evolution of SLD and on the definition of the cognitive profile.

Furthermore, the rehabilitation and therapeutic history of the participants was not considered in the present research. These factors could represent a source of variability, as therapeutic interventions may have influenced the cognitive and academic performance detected at the time of the assessment. Therefore, there is a need to collect detailed information on previous and ongoing interventions, considering duration, frequency, initiation period, and specific treatment goals, to further investigate whether and to what extent they can act as moderators of assessment outcomes.

Further studies should deepen these links through larger samples and advanced school methodologies, allowing to identify clusters between cognitive processes and learning difficulties. This approach could also be crucial for extending analysis to preschool age, promoting early preventive interventions (Capobianco, 2025), for the development of more effective and personalized rehabilitation protocols. Hence, future research should inquire about the possibility of further different functional patterns, specifically including the intraparietal lobe, in school-aged girls diagnosed with SLD; other eligible regions of interest include the dl-PFC, and more profound areas, such as bilateral angular gyrus (AG), anterior cingulate cortex (ACC), and fusiform gyrus (FFG).

## 5 Conclusion

Results confirm the multidimensional nature of Specific Learning Disabilities, highlighting variable relationships between cognitive profiles and academic skills as a function of individual development and differences. In detail, working memory emerges as a central and transversal component, implicated in multiple learning domains.

Reading comprehension is the most associated skill with WISC-IV indices, confirming the importance of verbal skills in learning processes. Moreover, observed gender differences suggest distinct functioning patterns, with a greater involvement of working memory in females, especially in logical-mathematical skills.

Evidence from neuroimaging studies contribute to the interpretation of these findings, showing differences in levels of neural network connectivity associated with different cognitive profiles. Specifically, gender-related morphological differences, especially observed in girls, are consistent with the predictive role of Verbal Comprehension (VCI) and Working Memory (WMI) indices. Alterations in the parietal lobe, particularly in the intra-parietal sulcus, both in terms of gray-white matter ratio and functional connectivity, could help explain emerged findings, especially in mathematical skills.

This study is innovative as it allows us to derive results from clinical data exploration, collected within a facility dealing with evaluation and intervention at developmental age. The main purpose of this study was to analytically explore, from a clinical database of 160 kids with SLD aged 7 to 14 years old, possible links between underlying processes, such as memory, processing speed, visuo-perceptual reasoning, and language skills – assessed through WISC-IV subtests – and the different learning skills that may be deficient in children with SLD between primary and lower secondary school. This type of analysis is still relatively underexplored in literature. Although numerous studies have investigated cognitive profiles in SLD through WISC-IV, such research predominantly focused on compound indices or profile patterns between clinical groups (Poletti et al., 2018; Giofrè et al., 2022), emphasizing differences in specific subtests but without systematically deepening into the relation with individual learning skills. Alongside, different research lines have examined the role of specific cognitive processes, such as working memory, in relation to school skills (Cornoldi et al., 2014; Toffalini et al., 2017). However, such contributions tend to focus on narrow learning domains, such as reading or mathematical skills, analyzing specific and isolated relationships.

More rarely, an integrated approach is adopted that simultaneously considers multiple WISC-IV subtests and a broad set of academic skills. Additionally, these analyses are often conducted in experimental settings or on individual aspects of cognitive functioning, rather than within complex clinical samples. The present findings result particularly relevant in prevention and early intervention. The ability to identify specific associations between cognitive processes and academic difficulties (Granato et al., 2025, 2026) allows for the design of more targeted rehabilitation or cognitive enhancement interventions, focused on underlying processes and not exclusively on deficit skills.

## Data Availability

The raw data supporting the conclusions of this article will be made available by the authors, without undue reservation.
